# The protective effects of *Ganoderma lucidum* polysaccharides on blood physiology, immune function, and organ architecture in growing stressed rabbits

**DOI:** 10.5455/javar.2025.l966

**Published:** 2025-09-24

**Authors:** Fatima S. Alaryani

**Affiliations:** Department of Biological Sciences, College of Science, University of Jeddah, Jeddah, Saudi Arabia.

**Keywords:** Ganoderma lucidum polysaccharides, heat stress, rabbits, pro-inflammatory cytokines

## Abstract

**Objective:**

This experiment explored the plausible effects of *Ganoderma lucidum* polysaccharides (GLP) to reduce the deterioration effects of heat stress (HS) in growing rabbits by studying blood physiology, growth, immunity, inflammation, and organ structure.

**Materials and Methods:**

Growing male rabbits (*n* = 160) were divided into 4 groups and fed a basal diet containing 0 (GLP0), 100 (GLP100), 250 (GLP250), and 400 (GLP400) mg of GLP/kg diet under tropical environmental conditions for 8 weeks. Growth, blood indices, redox state, immune markers, and histology of the liver and kidney were assessed.

**Results:**

The addition of GLP (100–400 mg/kg diet) significantly improved the growth indices and reduced the value of the feed conversion ratio (FCR) compared to the GLP0 group (*p < *0.05). The liver enzymes, cytochrome C and *caspase-3,* were significantly decreased by GLP supplementation, while it significantly improved IgG and IgM compared to the control group (*p < *0.05). Adding 250 or 400 mg of GLP significantly improved antioxidant enzymes and reduced oxidative stress markers compared to other groups (*p < *0.001). Supplementing diets with GLP up to 400 mg/kg diet had lower pro-inflammatory cytokines and greater *IL-10* compared to stressed rabbits in the GLP0 group (*p < *0.001). The renal and lung tissues were supported by the supplementation of GLP to the stressed rabbit diets.

**Conclusion:**

Overall, adding GLP to the diet can be recommended as an effective intervention to alleviate the adverse influences of HS. It enhances growth indices, maintains organ histology, boosts immunity, and reduces pro-inflammatory cytokines and apoptotic biomarkers.

## Introduction

Climate change has resulted in more frequent and intense heat waves during summer, which have a negative impact on both human and animal health [1]. Heat stress (HS), a direct consequence of climate change, poses a significant threat to the food supply chain, particularly within the livestock industry. Rabbits, being popular animals known for their high-quality meat and fur [2], as well as their superior reproductive capacity, are a preferred choice for farmers and other stakeholders [2]. Rabbit meat is especially favored by individuals with cardiovascular, diabetic, and hypercholesterolemic conditions due to its low cholesterol content and high protein levels [2,3]. However, rabbits have limited sweat glands, making them susceptible to high temperatures, especially in the summer months. Since most rabbits are bred in tropical and subtropical regions, their productivity often decreases or stops completely during these periods [4,5]. Scientists have conducted extensive research over the last years on the adverse consequences of HS on the physiological functions of animals, including rabbits [6–9].

HS can reduce the availability of nutrients, cause malabsorption [6], impair blood hemostasis, and cause hormonal imbalance [10] in rabbits. Moreover, HS negatively influences feeding activities [11], growth [12], immunity [13], and the survival of growing rabbits [9]. Moreover, HS induces the generation of oxidative stress, triggers inflammation, causes DNA damage, promotes apoptosis, and leads to mitochondrial dysfunction [14], weakening the functionality and structure of organs [12,13].

Consequently, rising temperatures notably decline the productivity of rabbit farms in providing meat and thus threaten food security.

Numerous studies have explored various strategies, including dietary interventions, to mitigate the detrimental influences of HS on growing rabbits. Research has shown that dietary supplementation can reduce the inflammatory response triggered by HS [7,13,14]. This is achieved by modulating the immune response, decreasing inflammation, and preserving testicular structure and sperm production, ultimately improving reproductive performance in rabbits exposed to hot environments.

*Ganoderma lucidum* (GL) is a group of fungi belonging to the *Basidiomycota* phylum. Widely distributed across subtropical, tropical, and temperate regions of America, Europe, Africa, and Asia, GL primarily consists of ash, carbohydrates, fats, fibers, and proteins [15]. GL contains a variety of bioactive compounds, such as triterpenes, polysaccharides, fatty acids, steroids, nucleosides, amino acids, proteins, alkaloids, and inorganic constituents. *Ganoderma lucidum* polysaccharide, a key bioactive component extracted from GL spore powder or fruiting bodies, is a natural biomacromolecule. Its beneficial impacts on human health have led to its widespread use in food, medicine, and health products [16,17]. GLP has been recognized for its diverse health benefits [18], including antioxidant, immunomodulatory, anti-tumor, anti-cancer, anti-diabetic, anti-obesity, and gut microbiota-regulating properties. Given GLP’s potent biological activities, we suggested that dietary supplementation could enhance rabbit thermotolerance by stimulating antioxidant defenses, bolstering immunity, and mitigating inflammatory and apoptotic processes. Hence, this research investigated the benefits of dietary addition of GLP on the growth, feed efficiency, blood physiology, immune ability, redox regulation, and inflammatory cytokines in fattening rabbits under HS environments.

## Material and Methods

### Ethical approval

The study protocol was reviewed and approved by the University of Jeddah, following the ethical standards outlined in the U.K. Animals (Scientific Procedures) Act of 1986, the EU Directive 2010/63/EU for animal experimentation, and the National Research Council’s Guide for the Care and Use of Laboratory Animals [8023]. All procedures adhered to the ARRIVE guidelines.

### Animals and housing conditions

*Ganoderma lucidum* polysaccharides (GLP; CAS: 223751–82–4) were obtained from NOVO (Hefei, Anhui, China). A total of 160 healthy male rabbits (average weight: 655 ± 4.25 gm, age: 5 weeks) were enrolled. Animals were randomly divided into four groups, with 20 replicates per group and two rabbits per replicate. All groups were kept under the same natural environmental conditions. The control group (GLP0) received a standard basal diet (Table 1), while the other three groups were supplemented with 100 mg (GLP100), 250 mg (GLP250), or 400 mg (GLP400) of GLP per kg of feed for eight weeks. The rabbits were housed individually in galvanized wire cages (50 × 40 × 30 cm) equipped with feeders and nipple drinkers. Before the trial began, the diet was prepared and pelleted to meet the nutritional requirements for growing rabbits according to Blas and Mateos [19]. Feed and water were available *ad libitum*.

All rabbits were housed in a naturally ventilated facility under uniform management and hygiene conditions. To assess environmental HS, ambient temperature and relative humidity were recorded using an automatic thermo-hygrometer (Wertheim, Germany). The temperature–humidity index (THI) was calculated as described by Marai et al. [20]:

THI = dp − [(0.31 − 0.31 × (RH/100)) × (dp − 14.4)]

where dp is the dry bulb temperature in °C. THI values were categorized as follows: > 30.0 (very severe HS), 29.0–30.0 (severe HS), 27.8–28.9 (moderate HS), and < 27.8 (no HS).

### Growth performance and blood sampling

Body weight and feed intake were measured every four weeks. From these data, average daily gain (ADG) and feed conversion ratio were calculated. At the end of the trial, ten rabbits per group were randomly selected for blood sampling from the marginal ear vein using sterile syringes, following the procedure of Massányi et al. [21]. Blood was collected without anticoagulants and kept at room temperature for 2 h to allow clotting. The samples were then centrifuged at 3,500 rpm for 20 min, and the serum was stored at −20°C for further analysis.

### Metabolic, antioxidant, and immune parameters

Serum levels of total bilirubin (TB), creatinine, urea, triglycerides (TG), lactate dehydrogenase (LDH), and gamma-glutamyl transferase (GGT) were determined using commercial kits from Bio Diagnostic Co. (Giza, Egypt). Antioxidant enzyme activities—catalase (CAT), superoxide dismutase (SOD), and glutathione peroxidase (GPx)—were measured with kits from BioMérieux (Marcy-l’Étoile, France) as per the manufacturer’s protocols [22]. Lipid peroxidation (MDA) and protein oxidation (protein carbonyl) were assessed according to Eid et al. [23] using Abcam kits. Immunoglobulin levels of IgG (SKU: RGG71–K01) and IgM (SKU: RGM71–K01) were evaluated using ELISA kits for rabbits (Eagle Biosciences, Amherst, MA, USA). Assay sensitivities were 1.920 ng/ml (IgG) and 1.684 ng/ml (IgM), with dynamic ranges of 7.81–500 ng/ml and 6.25–200 ng/ml, respectively.

**Table 1.** Components and chemical analysis of the diet applied for feeding growing rabbits.

Items	Control diet
Ingredients (gm/1000gm DM)	
Soybean meal 44%	180
Berseem hay	310
Molasses	20
Wheat bran	190
Maize grains	180
Barley grains	120
Limestone	10
NaCl	5
Premix*	5
Chemical analysis (%, on DM basis)	
Crude fiber	13.48
Dry matter	85.81
Ash	5.10
Organic matter	94.90
Crude protein	17.20
Ether extract	2.68
Metabolized energy	1942 Kcal/kg

*Each kg of premix (minerals and vitamins mixture) contains vitamins. vit. A, 20,000 IU; D3, 15,000 IU; vit. E, 8.33 gm; vit. K, 0.33 gm; vit. B2, 1.0 gm; vit. B1, 0.33 gm; B6, 0.33 gm; vit. B12, 1.7 mgm; vit. B5, 8.33 gm; vit. folic acid, 0.83 gm; biotin, 33 mg; choline chloride, 200 gm; Cu 0.1 mg, Fe 75.0 mg, pantothenic acid, 3.33 gm; iodine 0.2 mg, Co 0.5 mg, Mg 8.5 mg, Mn 8.5 mg, ZnO 20 mg, 0.1 mg sodium selenite. The diet of all experimental groups was isonitrogenous and isocaloric.

### Inflammatory and apoptotic markers

Levels of *Interleukin–6* (*IL–6*, Code: E–EL–RB0014), *Interferon–gamma* (*IFN–γ*, Code: E–EL–RB0679), and *Interleukin–10* (*IL–10*, Code: E–EL–RB0487) were analyzed using sandwich ELISA kits from Elabscience (Houston, TX, USA). Nuclear factor–κB (NF–κB, Code: MBS722751) and cytochrome C (CYTO, Code: MBS285651) were measured using competitive ELISA kits from Mybiosource (San Diego, CA, USA). Nitric oxide levels were assessed following Żurawiński et al. [24]. Lysosomal activity was evaluated as described by [25]. *Caspase-3* (Code: AB39401) was measured using a colorimetric ELISA kit from Abcam (China).

### Histological examination

Three rabbits per group were euthanized, and their lungs and kidneys were collected. Tissues were fixed in 10% buffered formalin for three days, with daily replacement of the fixative. Samples were dehydrated in increasing ethanol concentrations (30–100%), embedded in paraffin, sectioned at 3–5 µm, and stained with hematoxylin and eosin (H&E) [26]. Slides were examined and photographed using an Olympus CX31 microscope equipped with a DP72 digital camera and imaging software.

**Figure 1.**
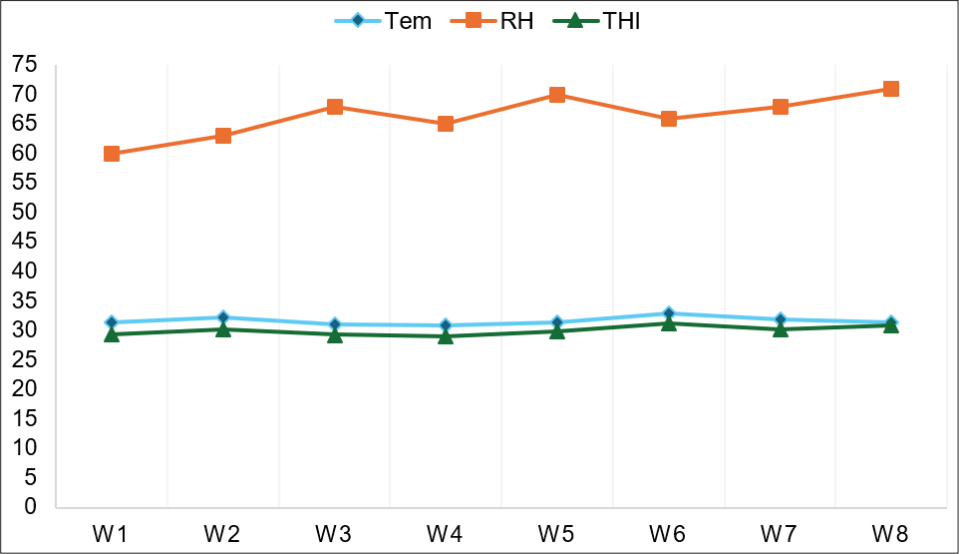
The THI values, relative humidity (RH, %), and ambient temperature (Temp, °C) throughout the experimental period in the natural environment.

### Statistical analysis

Data normality was confirmed using the Shapiro–Wilk test. One-way ANOVA followed by post hoc testing was used to compare means across groups. Statistical analyses were performed using IBM SPSS Statistics v26. Results are presented as mean ± standard error (SE), and differences were considered statistically significant at *p *< 0.05.

## Results

### Temperature–humidity index (THI)

As shown in Figure 1, the values were 29.38 and 30.96 at the first and last weeks of the study, indicating severe HS in growing rabbits. The data on THI revealed that the ambient temperature and relative humidity were 31.5°C and 60% at the beginning of the study. By the end of the study, the ambient temperature had remained at 31.5°C, but the relative humidity had increased to 71%.

### Growth performance

The impact of dietary inclusion of GLP on the growth metrics of stressed growing rabbits is presented in Table 2. Over the 4-week treatment period, rabbits fed 400 mg of GLP showed greater BW compared to other groups (*p < *0.001). The BW at 8 weeks of treatment was highest in rabbits fed 250 or 400 mg of GLP per kg of diet. Stressed rabbits fed GLP had a better final BW (0–8 weeks) than the GLP0 group (*p < *0.001). During the 4–8-week period, weight gain (WG) was significantly improved compared to the GLP0 group (*p < *0.001). HS significantly decreased FI compared to the treated groups (*p < *0.001) throughout the study period, with the maximum FI observed in the GLP400 group (*p < *0.001). FCR was not affected by the addition of GLP (*p > *0.05).

### Liver and kidney functions

Creatinine decreased significantly only in the GLP250 group (*p < *0.001), while the other groups had similar results (*p > *0.05) (Table 3). Rabbits fed 100 mg of GLP had the lowest urea levels compared to other groups (*p = *0.015). Feeding stressed rabbits with GLP resulted in lower levels of TB than the stress group (GLP0). TG and LDH were significantly reduced by GLP supplementation (except LDH in GLP100; *p < *0.001). Rabbits in the GLP250 and GLP400 groups had the lowest TG and LDH levels. Dietary inclusion of GLP induced a significant decrease in GGT levels in a dose-dependent manner (*p < *0.001).

### Redox regulation and immunoglobulins

Supplementation with 250 or 400 mg of GLP significantly increased SOD levels compared to other groups (*p < *0.001) (Table 4). GPx levels were highest in the GLP400 group (*p < *0.001), while rabbits in the GLP250 and GLP100 groups had higher GSH levels than the stressed rabbits (*p < *0.001). The treatments had no impact on CAT levels (*p = *0.35). High levels of oxidative stress biomarkers (MDA and PC) were triggered by HS in rabbit serum, but the addition of GLP reduced this elevation (*p < *0.001). Feeding GLP resulted in a significant increase in the serum levels of IgG compared to the untreated group (*p < *0.001). IgM levels were higher in rabbits fed a 250 or 400 mg/kg diet compared to other treatments (*p < *0.001), while GLP100 also increased IgG levels compared to the GLP0 group (*p < *0.001).

### Pro-inflammatory cytokines and apoptosis markers

The impacts of dietary GLP on pro-inflammatory cytokines and apoptosis markers of stressed rabbits are exhibited in Table 5. Rabbits fed diets with supplemental GLP showed a significant reduction in *IFN–γ* (*p = *0.003) and *IL–6* (*p < *0.001) compared to the GLP0 group. The *IL-10* levels in the serum of stressed rabbits fed GLP were higher than those in the HS group (GLP0, *p = *0.004). NO was significantly heightened in a dose-dependent way by gradually increasing GLP in the rabbit diets (*p < *0.001). LA was the highest in the GLP250 and GLP400 groups (*p < *0.001). HS led to a significant increase in NF–κB levels, while the dietary inclusion of GLP reduced them by 31.8% (GLP100), 28.2% (GLP250), and 27.9% (GLP400). Rabbits fed 250 or 400 mg of GLP/kg diet showed a substantial decline in *Caspase-3* levels, while there was a significant increase in CYTO levels compared to the GLP0 and GLP100 groups. Overall, HS resulted in a considerable increase in pro-inflammatory markers (*IL-6,* NF-κB, and *IFN*-γ) and a significant reduction in anti-inflammatory markers (*IL-10)* and apoptosis (CYTO and *Caspase*-*3*). However, dietary inclusion of GLP can target the inflammatory/apoptosis pathways in stressed rabbits.

### Histological findings

**Figure 2.**
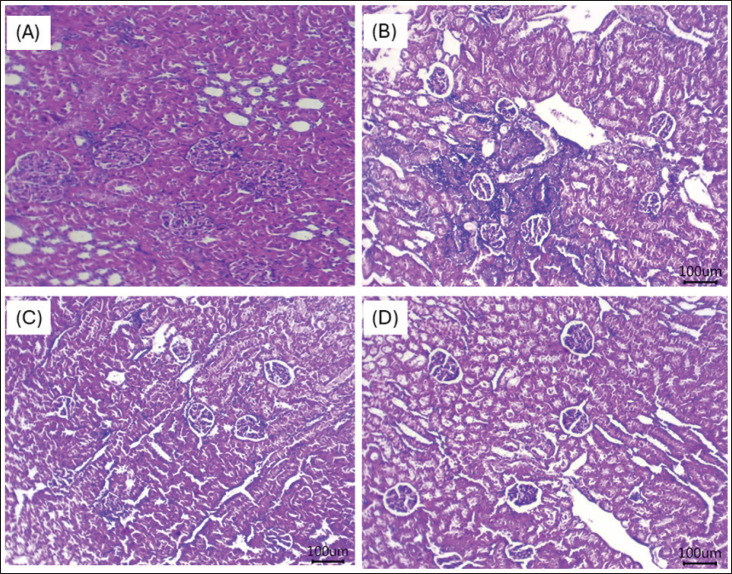
(A–D) Representative photomicrographs of kidney tissues from growing rabbits reared under HS and fed various levels of GLP 0 (Fig. 2A), 100 (Fig. 2B), 250 (Fig. 2C), and 400 (Fig. 2D) mg/kg diet. Rabbits kept under HS had mild fibrosis with moderate tubular damage, intra-tubular aggregation of sloughed epithelium, moderately congested glomeruli, and atrophies (Fig. 2A). In the rabbit group fed 100 mg/kg (Fig. 2B), there was mild cortical interstitial nephritis, separated deteriorated tubules, and a remarkably atrophied glomerulus. The growing rabbits fed 250 (Fig. 2C) and 400 (Fig. 2D) exhibited regular histological structures of their glomeruli, tubules, and interstitial tissue.

**Figure 3.**
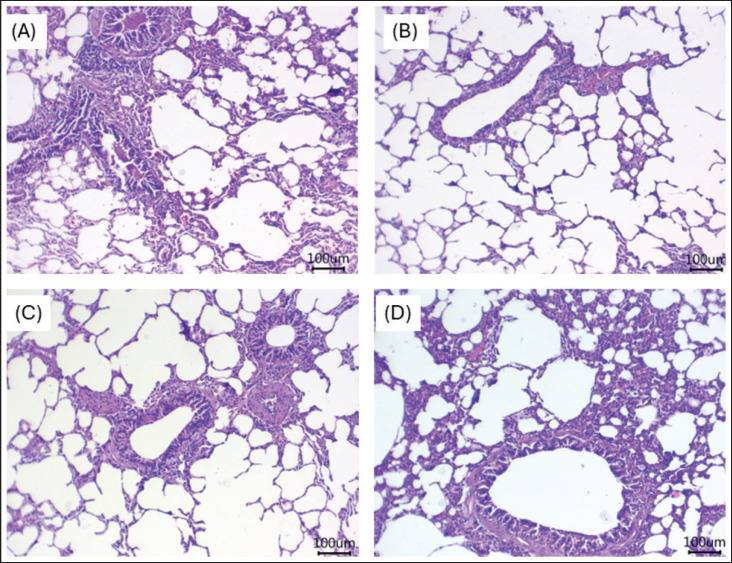
(A–D) Representative photomicrographs of lung tissues from growing rabbits reared under HS and fed various levels of GLP 0 (Fig. 3A), 100 (Fig. 3B), 250 (Fig. 3C), and 400 (Fig. 3D) mg/kg diet. HS can induce the accumulation of inflammatory cells and disrupt the alveolar architecture, leading to diffuse damage across alveoli, alveolar sacs, alveolar septa, and bronchi (Fig. 3A). The addition of GLP at concentrations of 100 (Fig. 3B), 250 (Fig. 3C), and 400 (Fig. 3D) improved lung architecture by reducing inflammatory cell infiltration and preserving the integrity of alveoli, alveolar sacs, alveolar septa, and bronchi.

The impacts of dietary supplements with GLP on the kidney and lung tissues of growing rabbits are presented in Figures 2 and 3, respectively. Microscopically, rabbits kept under HS had mild fibrosis with intra-tubular aggregation of sloughed epithelium, moderately congested glomeruli, moderate tubular injury, and atrophy (Fig. 2A). In the rabbit group fed 100 mg/kg (Fig. 2B), there was mild cortical interstitial nephritis, separated deteriorated tubules, and a remarkably atrophied glomerulus. The growing rabbits fed 250 (Fig. 2C) and 400 (Fig. 2D) exhibited regular histological structures of their glomeruli, tubules, and interstitial tissue. HS can induce the gathering of inflammatory cells and disrupt the alveolar architecture, leading to diffusing damage across alveoli, alveolar sacs, alveolar septa, and bronchi (Fig. 3A). The addition of GLP at concentrations of 100 (Fig. 3B), 250 (Fig. 3C), and 400 (Fig. 3D) improved lung architecture by reducing inflammatory cell infiltration and preserving the integrity of alveoli, alveolar sacs, alveolar septa, and bronchi.

**Table 2.** Influence of various levels of dietary supplemental *Ganoderma lucidum* polysaccharides (GLP) on growth performance of rabbits subjected to heat stress.

Variable	*Ganoderma lucidum* polysaccharides (GLP) level				*p*-value
	GLP0	GLP100	GLP250	GLP400	
Body weight (BW, gm)					
Initial body weight	655.5 ± 4.25	655.00 ± 3.80	660.00 ± 3.83	659.20 ± 3.98	0.764
BW at 4 weeks	1477.00 ± 15.19^b^	1487.50 ± 17.88^b^	1469.50 ± 18.14^b^	1528.50 ± 6.19^a^	0.042
BW at 8 weeks	2020.00 ± 16.65^c^	2073.00 ± 18.38^b^	2111.00 ± 16.83^a^	2131.50 ± 11.62^a^	0.0001
Body gain (WG, gm)					
WG, 4 weeks	821.50 ± 15.37	832.50 ± 17.61	810.00 ± 18.17	869.00 ± 5.86	0.050
WG, 4–8 weeks	543.00 ± 8.03^d^	585.50 ± 9.73^c^	641.50 ± 15.31^a^	603.00 ± 13.42^b^	<0.001
WG, 0–8 weeks	1364.50 ± 17.38^b^	1418.00 ± 17.03^a^	1451.50 ± 16.90^a^	1472.00 ± 11.67^a^	<0.001
Feed intake (FI, gm)					
FI, 4 weeks	2386 ± 41.34^d^	2430 ± 47.07^b^	2.343 ± 47.89^c^	2552 ± 17.75^a^	0.005
FI, 4–8 weeks	1697 ± 29.37^d^	1809 ± 22.48^c^	1900 ± 42.56^b^	2012 ± 44.44^a^	<0.001
FI, 0–8 weeks	4083 ± 49.44^d^	4239 ± 46.41^c^	4355 ± 40.75^b^	4458 ± 41.79^a^	<0.001
Feed conversion ratio (FCR, gm feed /gm gain)					
FCR, 4 weeks	2.91 ± 0.01	2.92 ± 0.01	2.89 ± 0.01	2.94 ± 0.02	0.094
FCR, 4–8 weeks	3.13 ± 0.03	3.09 ± 0.03	3.14 ± 0.03	3.15 ± 0.01	0.377
FCR, 0–8 weeks	2.99 ± 0.01	2.99 ± 0.02	3.00 ± 0.01	3.03 ± 0.02	0.246

^a, b, c, d,^ means within a row without a common superscript differ at *p < *0.05.

^1^ GLP0, GLP100, GLP250, and GLP400 = 0, 100, 250, and 400 mg *G. lucidum* polysaccharides (GLP)/kg DM diet, respectively.

**Table 3.** Influence of various levels of dietary supplemental *G. lucidum* polysaccharides (GLP) on blood biochemistry of growing rabbits subjected to heat stress.

Item	*Ganoderma lucidum* polysaccharides (GLP) level				*p* values
	GLP0	GLP100	GLP250	GLP400	
Creatinine, mg/dl	2.84 ± 0.04^a^	1.37 ± 0.03^b^	1.36 ± 0.04^b^	1.25 ± 0.02^c^	<0.001
Urea, mg/dl	71.13 ± 0.58^a^	61.84 ± 4.19^b^	71.98 ± 1.25^a^	73.46 ± 1.2^a^	0.015
TB, mg/dl	0.97 ± 0.01^a^	0.74 ± 0.02^b^	0.72 ± 0.02^b^	0.76 ± 0.01^b^	<0.001
TG, mg/dl	74.41 ± 2.74^a^	68.86 ± 0.70^b^	49.91 ± 0.73^c^	50.27 ± 1.46^c^	<0.001
LDH, U/l	26.69 ± 0.63^a^	27.92 ± 1.13^a^	24.07 ± 0.32^b^	23.21 ± 0.38^b^	0.001
GGT, U/l	43.56 ± 1.00^a^	29.36 ± 0.64^b^	24.31 ± 0.46^c^	20.93 ± 0.49^d^	<0.001

Total glycerides (TG), lactate dehydrogenase (LDH), gamma-glutamyl transferase (GGT), and total bilirubin (TB). ^a, b, c^ Means within a row without a common superscript differ at *p < *0.05.^1^GLP0, GLP100, GLP250, and GLP400 = 0, 100, 250, and 400 mg *Ganoderma lucidum* polysaccharides (GLP)/kg DM diet, respectively.

## Discussion

Rabbits have recently become important in meat production worldwide and have the potential to help alleviate poverty. However, these animals are highly sensitive to high temperatures, which can negatively impact their growth, productivity, and reproductive capacity during hot seasons. HS is a major environmental concern associated with global climate change. Using natural substances to enhance rabbit health, welfare, and growth could be an effective strategy to mitigate the detrimental effects of HS. This study evaluated the effects of dietary GLP inclusion on growth, organ histology, blood physiology, immune function, pro-inflammatory cytokines, and apoptosis indicators in rabbits under changing environmental stress conditions. This is the first study to explore the protective effect of GLP against HS-induced blood imbalance, growth decline, immune dysfunction, redox imbalance, and inflammatory promotion in growing rabbits. Our findings suggest that *G. lucidum* polysaccharides (GLPs) can alleviate the adverse effects of HS by reducing inflammation, oxidative stress, and modulating immune response. GLPs also enhance antioxidant capacity, improve mitochondrial function, and boost anti-inflammatory mediators, as evidenced by their ability to maintain organ architecture.

**Table 4.** Influence of various levels of dietary supplemental *G. lucidum* polysaccharides (GLP) on redox homeostasis and immunoglobulins of growing rabbits subjected to heat stress.

Item	*Ganoderma lucidum* polysaccharides (GLP) level				*p* values
	GLP0	GLP100	GLP250	GLP400	
Antioxidative markers					
SOD, nmol/ml	0.19 ± 0.03^b^	0.22 ± 0.03^b^	0.78 ± 0.01^a^	0.78 ± 0.02^a^	<0.001
GPx, nmol/ml	0.21 ± 0.03^c^	0.28 ± 0.01^b^	0.30 ± 0.01^b^	0.41 ± 0.01^a^	<0.001
CAT, nmol/ml	0.19 ± 0.03	0.21 ± 0.02	0.19 ± 0.01	0.16 ± 0.001	0.350
Oxidative stress indices					
PC, μmol/ml	1.34 ± 0.05^a^	0.17 ± 0.01^b^	0.15 ± 0.01^b^	0.12 ± 0.00^b^	<0.001
MDA, μmol/ml	3.21 ± 0.03^a^	1.23 ± 0.04^b^	1.17 ± 0.04^b^	1.15 ± 0.04^b^	<0.001
Immunoglobulins					
IgG, ng/ml	28.51 ± 0.95^b^	54.16 ± 0.84^a^	53.46 ± 0.90^a^	53.27 ± 1.08^a^	<0.001
IgM, ng/ml	40.61 ± 1.23^c^	77.56 ± 0.52^b^	79.48 ± 1.24^a^	80.47 ± 0.73^a^	<0.001

Protein carbonyl (PC), superoxide dismutase (SOD), catalase (CAT), glutathione peroxidase (GPx), malondialdehyde (MDA), immunoglobulin G (IgG), and immunoglobulin M (IgM). ^a, b^ means within a row without a common superscript differ at *p < *0.05.

^1^GLP0, GLP100, GLP250, and GLP400 = 0, 100, 250, and 400 mg *G. lucidum* polysaccharides (GLP)/kg DM diet, respectively.

**Table 5.** Influence of various levels of dietary supplemental *G. lucidum* polysaccharides (GLP) on inflammatory cytokines and apoptosis markers of growing rabbits subjected to heat stress.

Item	*Ganoderma lucidum* polysaccharides (GLP) level				*p* values
	GLP0	GLP100	GLP250	GLP400	
*IL–6*, pg/ml	25.89 ± 2.93^a^	14.00 ± 1.18^b^	11.43 ± 0.49^b^	11.96 ± 0.47^b^	< 0.001
*IFN–γ*, pg/ml	27.40 ± 3.65^a^	16.93 ± 1.33^b^	16.34 ± 0.36^b^	14.86 ± 0.49^b^	0.003
*IL–10*, pg/ml	46.65 ± 11.22^b^	78.48 ± 0.74^a^	77.67 ± 1.38^a^	77.47 ± 0.69^a^	0.004
NO, μmol/l	0.22 ± 0.01^d^	0.49 ± 0.04^c^	0.92 ± 0.07^b^	1.16 ± 0.01^a^	< 0.001
LA, ug/ml	1.45 ± 0.05^c^	2.52 ± 0.07^b^	3.33 ± 0.07^a^	3.24 ± 0.05^a^	< 0.001
NF–κB, ng/ml	120.15 ± 10.49^a^	81.86 ± 0.64^d^	86.25 ± 2.39^b^	83.52 ± 0.80^c^	0.001
CYTO, μg/ml	0.19 ± 0.01^c^	0.45 ± 0.14^b^	1.21 ± 0.03^a^	1.14 ± 0.02^a^	< 0.001
*Caspase-3*, ng/ml	11.75 ± 0.53^a^	4.52 ± 0.26^b^	1.32 ± 0.07^c^	1.37 ± 0.11^c^	< 0.001

Interleukin 6 (*IL–6*), interleukin 10 (*IL–10*), interferon gamma (*IFN–γ*), nuclear factor kappa B (NF–κB), nitric oxide (NO), lysosome activity (LA), and cytochrome c (CYTO).^a, b, c, d,^ means within a row without a common superscript differ at *p < *0.05.^1^GLP0, GLP100, GLP250, and GLP400 = 0, 100, 250, and 400 mg *G. lucidum* polysaccharides (GLP)/kg DM diet, respectively.

Natural substances like GLPs are being considered as alternatives to antibiotics due to environmental concerns, bacterial resistance, and safety issues. *Ganoderma lucidum*, a type of mushroom, is known for its health benefits and contains various compounds such as triterpenoids, Ganoderic acids, lucinedic acids, ganodermic acids, lucidones, nucleotides, and polysaccharides, which contribute to its pharmaceutical and health-promoting properties.

The present study reveals that GLP stimulated the growth performance in rabbits exposed to hyperthermia conditions. Previous studies have confirmed that animals exposed to hyperthermia, such as rabbits, experience a significant decrease in growth due to disrupted feed intake, decreased release of digestive enzymes, and disturbed nutrient digestibility. Adding GLP to their diet improved growth due to its antioxidant and immunomodulatory effects. Polysaccharides are known for their ability to modify nutrient absorption, potentially providing health benefits such as enhanced glycemic control and increased satiety. Moreover, polysaccharides isolated from *Laminaria japonica* also improved the growth in pigs by promoting the secretion of digestive enzymes (amylase and lipase activities) and improving the health of the intestinal canal [27]. Others suggested that GLP might be due to corrected digestion and bone accretion, thus having a confirmed release on animal growth [28]. Similarly, GL (10 gm/kg diet) enhanced the growth performance in growing rabbits [29]. The study by Wang et al. [17] found that GLP can regulate body weight in rats induced by a high-fat diet (HFD) and hyperlipidemia. This supports the capability of GLP to sustain the organ structure. In line with our results, Qin et al. [30] found that including GLP in the diet (100 mg/gm diet) significantly promoted the growth of rabbits by supporting cecal fermentation variables and the composition and structure of intestinal flora. Promoting rabbit growth during the HS conditions by enriching diets with GLP is attributed to their antimicrobial and antioxidant properties.

Polysaccharides are active compounds in GL that have various pharmacological properties, such as immunomodulatory, antitumor, cardiovascular, and antihepatotoxic properties. However, their protective effects against the negative impacts of HS remain unexplored. We suggest that GLP can regulate organ functions and support animal health after challenges with environmental stress. Blood biochemistry is a mirror of the health and well-being of an animal. HS can induce significant changes in the physiological pathways in the animal body. We observed that HS induced higher levels of liver enzymes (LDH and GGT), lipid profile (TG), and kidney markers (creatinine and urea) in growing rabbits. The GLP administration could sustain the functions of the liver and kidneys of growing rabbits under challenging environmental stress. GLP also has antioxidant effects that can promote growth in rabbits. The hepatoprotective effects of GLP have been confirmed by several authors, as it reduces liver enzymes induced by environmental toxic agents such as CCl_4_ [16] and tert–butyl hydrogen peroxide. This action is attributed to several factors: [1] GLP’s potential to maintain hepatocytes and protect against oxidative stress; [2] GLP promotes the synthesis of GPx and SOD, supporting the body’s antioxidative response; [3] it can decrease oxidation in cell membranes, such as lipid or protein oxidation.

HS has been identified as an environmental factor that can stimulate the production of ROS. This is supported by the similar responses observed after HS and exposure to OS. Our study revealed that HS leads to higher levels of MDA and protein carbonyl (PC), as well as reduced antioxidant enzyme levels in the serum of stressed growing rabbits. The administration of GLP significantly decreased MDA and PC levels as observed in this study. OS occurs when there is an imbalance between ROS assembly and the body’s antioxidant defense mechanisms. An increase in ROS can exacerbate metabolic dysfunctions and even lead to cell death, as ROS can damage important cellular components such as lipids, proteins, and DNA. When ROS production exceeds the body’s antioxidant capacity, OS occurs. In such cases, robust antioxidants are needed to counteract the effects of OS. For example, GLP has shown potential in mitigating oxidative stress induced by metabolic disorders by supporting antioxidant enzymes [17]. GL also prevented the OS induced by heavy metals in poultry due to its antioxidant capacity. GL significantly promoted the antioxidant enzymes SOD and GSH–Px after broilers were exposed to heavy metals [31]. Moreover, Trebušak et al. [32] found that the dietary GL tended to reduce the lipid peroxidation in rabbit meat. The antioxidative function of GL is mainly due to its richness in polysaccharides [33].

Immunity declines in rabbits due to the harmful effects of the HS. However, supporting their health by fortifying diets with immunomodulatory agents can help the animals resist the HS. In this study, the concentrations of IgG and IgM were notably increased in all groups that received GLP, indicating an immunomodulatory effect of GLP. These results are consistent with previous findings by Abd El-Hack et al. [34], Ismail et al. [35], and Abdelnour et al. [36], who found that the addition of phytochemicals and symbionts improved the immune markers in rabbits.

GLP has been shown to boost both adaptive and innate immunity [18] by stimulating the production of anti-inflammatory cytokines such as *IL-10*. Promoting anti-inflammatory mediators in the body during adverse environmental conditions may represent a critical strategy to support health and reduce inflammation and apoptosis. *IL-10* was improved by the administration of GLP in rabbits’ diets. Many environmental issues trigger inflammation and apoptosis events, such as heavy metals and HS. It was found that GL decreased the pro-inflammatory cytokines (*TNF-*α*,* *IL-1β*, and *IL-6*) and diminished the statement of apoptotic elements (*Caspase-3)* in broilers contaminated with heavy metals in their diets [31]. Research by Shen et al. has demonstrated that GLP therapy is effective in alleviating OS by activating the *Nrf2*/*HO–1* pathway. *Nrf2* is a crucial redox-sensitive transcription factor that plays a role in protecting tissues from OS. Studies have shown that overexpression of *Nrf2* can reduce hepatic lipid peroxidation and the transcription of lipogenic enzymes. As seen in our data, there are many natural compounds that can alleviate the inflammatory cytokines induced by HS in rabbits, such as biological nano selenium [8], phycocyanin [7], and prodigiosin [6]. Cytochrome c (CYTO) is a crucial component of the mitochondrial electron transport chain, playing a pivotal role in maintaining mitochondrial health [12]. Cytochrome c release into the cytoplasm is a marker for apoptosis, while its translocation to the extracellular space can induce inflammation [16]. Extracellular cytochrome c can act as a damage-associated molecular pattern (DAMP). DAMPs are molecules released from damaged or dying cells that signal the immune system. This activation leads to the release of pro-inflammatory cytokines, such as *TNF-*α, *IL-1β*, and *IL-6*, initiating an inflammatory response [18]. HS has been shown to induce mitochondrial dysfunction by disrupting the CYTO pathway, while feeding rabbits with GLP in their diets demonstrated a positive impact on mitochondrial function. This was evidenced by the support of CYTO and a concurrent reduction in apoptosis markers such as *Caspase-3*. Previous research has shown that *G*. *lucidum* extract can improve mitochondrial function in the aged rat brain [37], suggesting its potential therapeutic application in mitigating inflammation and apoptosis induced by challenging HS in animals. Targeting mitochondrial damage under HS conditions represents a promising novel therapeutic approach.

NF-κB is a pivotal transcription factor that plays a crucial role in orchestrating various cellular processes, including inflammation and immune responses [38]. During HS conditions, the expression of NF–κB significantly increased, indicating an activation of the inflammatory response. Zhang et al. [39] demonstrated that GLP attenuated acetaminophen-induced acute hepatic damage by exerting antioxidants and anti-apoptotic effects, likely through the inhibition of the NF-κB signaling pathway. Furthermore, GLP has been shown to effectively alleviate rheumatoid arthritis symptoms in rat models. This beneficial effect is believed to be mediated, at least in part, by the inhibition of both the NF–κB and mitogen-activated protein kinase (MAPK) signaling pathways, which are key players in the inflammatory cascade associated with rheumatoid arthritis [40].

Nitric oxide is a potent vasodilator, meaning it relaxes blood vessels and improves blood flow. In this study, we found that GLP supplementation significantly increased the levels of NhO in the blood, indicating improved blood flow to the skin [11]. This method may be more effective in other animals, depending on the heat dissipation through sweat glands. In rabbits, it may help dissipate heat by increasing blood flow to the ears. Another explanation suggests that GLPs have a cardioprotective effect, leading to a significant reduction in systolic and diastolic blood pressure and heart rate [41].

Lungs are very critical organs during the HS condition, responsible for respiration. As rabbits have few sweat glands, promoting lung health is very important in this adverse condition. HS damages the lungs, while GLP maintains lung structure against cadmium poisoning in mice [42]. A recent study demonstrated the renal-protective effect of cardamom essential oil [43]. The ability of phytochemicals to maintain a renal histological profile may explain their protective action against HS [6,7,10]. Further research using transcriptomics and proteomics during HS could provide valuable insights for developing effective strategies to mitigate HS in animals. Further investigation, such as genetic alterations, is necessary to confirm these findings and the significant improvement in some organ function. Second, the study did not evaluate the role of growth hormones and stress hormones, which should be considered in future research. This molecule possesses a robust antioxidant function, and we need further clarification to support this hypothesis, especially in growing animals, to reduce the mortality caused by HS.

## Conclusion

In summary, HS can reduce overall health and cause physiological imbalances in rabbits, resulting in decreased productivity and growth. Therefore, supplementing with 250 or 400 mg of GLP improved growth, feed efficiency, enhanced immunity (IgM and IgG), and maintained organ health. These improvements were attributed to the anti-inflammatory, antioxidant, and anti-apoptotic effects of GLP. Further research is needed to identify the key biological properties of active compounds in certain natural plants to support their use in commercial rabbit production.

## List of abbreviations

ADG, average daily gain; CAT, catalase; FCR, feed conversion ratio; GGT, gamma-glutamyl transferase; GL,* Ganoderma lucidum*; GLP,* Ganoderma lucidum* polysaccharides; GPx, glutathione peroxidase; HS, Heat stress; LA, Lysosomal activity; LDH, lactate dehydrogenase; SOD, superoxide dismutase; TB, total bilirubin; TG, triglycerides; THI, temperature–humidity index.
